# 3D β-Ni(OH)_2_ nanowires/RGO composite prepared by phase transformation method for superior electrochemical performance

**DOI:** 10.1038/s41598-019-47120-9

**Published:** 2019-07-25

**Authors:** Wenxiu He, Xingsheng Li, Shengli An, Tongjun Li, Yongqiang Zhang, Jinlong Cui

**Affiliations:** 1 School of Chemistry Chemistry and Chemical Engineering, Inner Mongolia University of Science and Technology, Baotou, 014010 China; 2 College of Physics and Material Science, Henan Normal University, Xinxiang, 453007 China

**Keywords:** Batteries, Nanowires

## Abstract

In order to improve the capacity and rate performance of nickel-metal hydride (Ni-MH) batteries, we proposed a simple phase transformation method to synthesis 3D β-nickel hydroxide nanowires/reduced graphene oxide (β-Ni(OH)_2_ NWs/RGO) composite. β-Ni(OH)_2_ nanowires with diameters of 20–30 nm and lengths up to several micrometers were decorated on RGO layers, forming 3D network structure. The 3D β-Ni(OH)_2_ NWs/RGO composite displayed a discharge capacity of 343.2 mAh/g at 0.2C, and the capacity values has almost no loss after 100th cycles. When the discharge rate was at 5C, the capacity reached a value of 272.7 mAh/g, and its capacity retention was 79.5%. It showed good capacity and rate performance as a positive material for Ni-MH batteries.

## Introduction

In recent years, the new energy electric vehicles and hybrid electric vehicles have been received great attention account of the rapid energy consumption and the environmental pollution^1,2^. However, the main factor limiting the development of new energy vehicles is energy storage systems (Ni-MH batteries, Lithium-ion batteries, supercapacitors, etc.)^3–7^. Therefore, the energy storage systems have been become a research hotspot. Ni-MH batteries are widely used in hybrid electric devices because of their high energy density, fast charging and discharging, low cost, safety, etc^8–10^. Moreover, Ni(OH)_2_ as positive material of Ni-MH batteries is an key factor affecting the capacity performance^11^. Nevertheless, the volume change of Ni(OH)_2_ is large in the rapid charging-discharging process, leading to the rapid capacity fade^12,13^. In order to improve the capacity performance of Ni(OH)_2_ electrode, significant research efforts have focused on the synthesis of nanostructured Ni(OH)_2_ with different structures, such as nanoflowers^14–17^, nanoplates^18–20^, nanobelts^21^, nanorods^22^, nanowires^23–26^. Among these structures, 1D nanostructured Ni(OH)_2_ is considered as an ideal positive material of Ni-MH batteries among these structures, because of its large surface areas and small volume change during charging-discharging process. Therefore, the preparation Ni(OH)_2_ nanowires with high structural stability is a hot topic for practical applications of Ni-MH batteries.

Owing to the unique physical and chemical properties of graphene, it can increase conductivity of Ni(OH)_2_ and effectively relieve its volume expansion^27,28^. Thus, many researchers use graphene to compensate the disadvantages of Ni(OH)_2_ as positive material of Ni-MH batteries. To date, many methods have been used to synthesize Ni(OH)_2_/graphene compounds, such as hydrothermal method^29^, template method^30^, and electrochemical deposition method^31^, etc. Nevertheless, α-Ni(OH)_2_ nanowires and non-nanowires α-/β-Ni(OH)_2_ are generally obtained by the hydrothermal approach, and the reduction process of graphene oxide to graphene needs to add reducing agent. For template method, the use of template agent and surfactant results in increased costs. The post-treatment process is complex and needs to remove the template agent. For electrochemical deposition method, it is necessary to precisely control the reaction conditions in the deposition process, such as temperature, pH value, current or voltage, etc. As a typical layered structure material, Ni(OH)_2_ contains two main crystalline structures, α- and β-Ni(OH)_2_ ^32^. β-Ni(OH)_2_ is consisted of close-stacked 2D Ni(OH)_2_ layers without intercalated species, and α-Ni(OH)_2_ is consisted of close-stacked 2D Ni(OH)_2_ layers with anions and water molecules^33^. Therefore, the theoretical specific capacity of α-Ni(OH)_2_ is higher than that of β-Ni(OH)_2_. However, α-Ni(OH)_2_ is not stable in alkaline medium and easily transformed into β-Ni(OH)_2_. 3D β-Ni(OH)_2_ NWs/RGO was synthesized by phase transformation method according to this property of Ni(OH)_2_. The prepared method of β-Ni(OH)_2_ NWs/RGO by phase transformation method was rarely reported previously.

The phase transformation method is quite simple due to their low cost, easy operation, safe, and environmentally friendly natures. In the phase transformation process, α-Ni(OH)_2_ was transformed into β-Ni(OH)_2_ at 180 °C for 30 min in a NaOH solution, and the morphology of nanowires was retained. In 3D β-Ni(OH)_2_ NWs/RGO composite, 1D β-Ni(OH)_2_ nanowires and 2D RGO sheets formed a stable 3D network structures that provided sufficient space and rapid transfer paths for ions and electrons^34^. The RGO sheets could enhance the conductivity of β-Ni(OH)_2_ nanowires and effectively alleviate the volume expansion during charging-discharging process, and the β-Ni(OH)_2_ nanowires could prevent the aggregation of the RGO sheets. Under the synergistic effects of β-Ni(OH)_2_ NWs and RGO sheets, the 3D β-Ni(OH)_2_ NWs/RGO composite exhibited a better electrochemical performance. β-Ni(OH)_2_ NWs/RGO composite displayed superior rate performance compared with α-Ni(OH)_2_ NWs/RGO and β-Ni(OH)_2_ nanoplates/RGO (β-Ni(OH)_2_ NPs/RGO) composites.

## Results and Discussion

**Figure 1:**
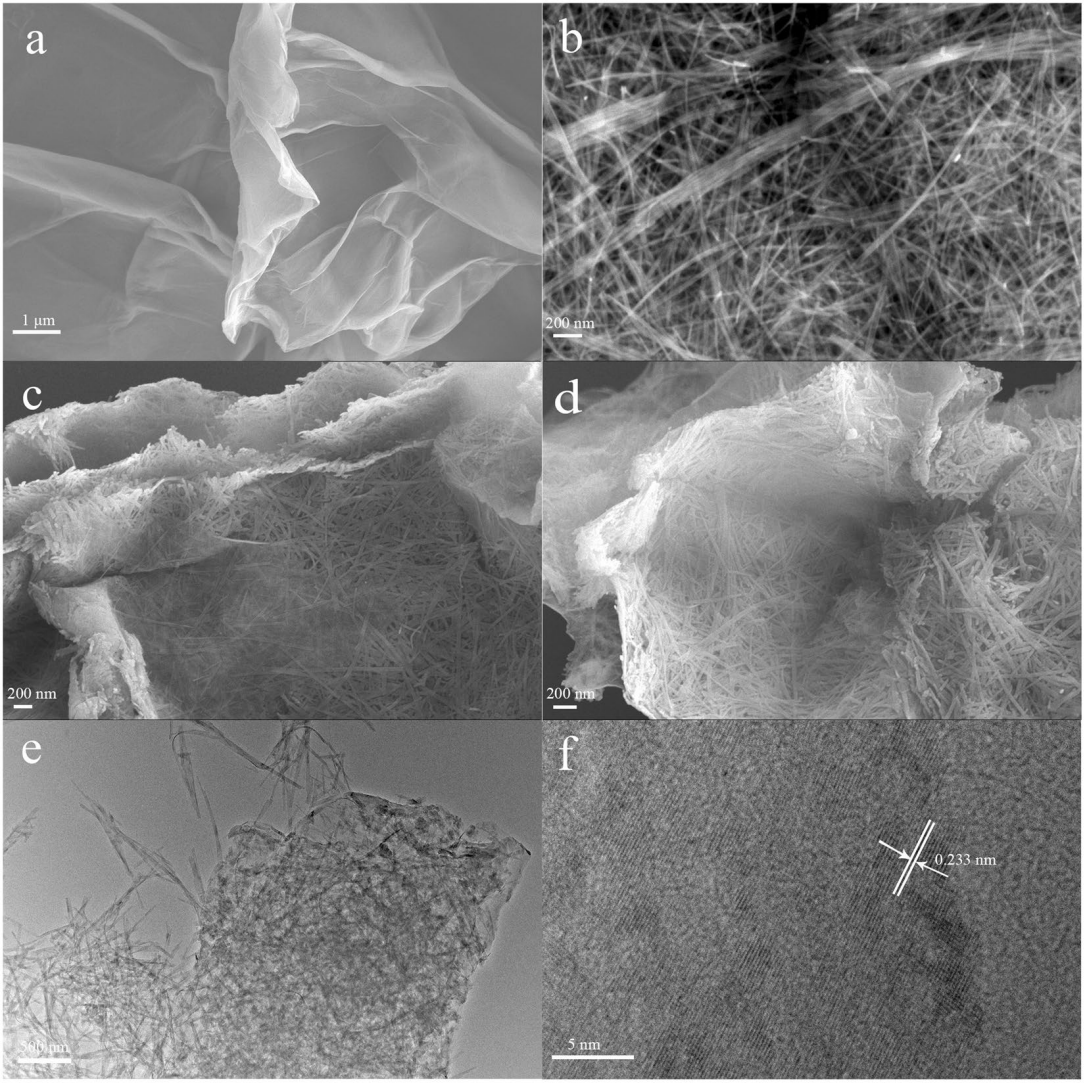
SEM images of GO (**a**), β-Ni(OH)_2_ NWs (**b**), α-Ni(OH)_2_ NWs/RGO (**c**), β-Ni(OH)_2_ NWs/RGO (**d,e**) TEM image and (**f**) HRTEM image of β-Ni(OH)_2_ NWs/RGO.

The morphology evolution process of α-Ni(OH)_2_ NWs/RGO composite in NaOH solution were characterized by SEM analysis. Figure 1a shows the SEM image of GO. It can be seen that GO sheets are like transparent voile with many wrinkles. Figure 1b exhibits the typical nanowires of β-Ni(OH)_2_, the average diameters were 20 nm. For α-Ni(OH)_2_ NWs/RGO composite, α-Ni(OH)_2_ nanowires with lengths up to several micrometers and diameters of about 30 nm were supported on RGO sheets to form 3D network structure in Fig. 1c. Figure 1d dispalys the morphology of β-Ni(OH)_2_ NWs/RGO composite after being hydrothermally treated for 30 min, and its morphology was still unchanged. The change of crystal phase has taken place in NaOH solution, and the nanowires morphology was maintained^35^. Compared with the pure β-Ni(OH)_2_ NWs, the diameter of β-Ni(OH)_2_ NWs becomes larger for β-Ni(OH)_2_ NWs/RGO composite, which is attributed to the addition of RGO. β-Ni(OH)_2_ NWs were uniformly loaded on RGO sheets (Fig. 1e). The lattice space of β-Ni(OH)_2_ NWs is 0.233 nm, which corresponds to (101) plane (Fig. 1f). It further demonstrates that the prepared materials are β-Ni(OH)_2_ NWs and RGO composites. The EDS elemental mapping image displays Ni, O and C elements, indicating that Ni(OH)_2_ NWs are uniformly dispersed on the whole RGO sheets (Fig. S1). The EDS analysis further suggests that the synthesized material is consisted of Ni(OH)_2_ and RGO. For β-Ni(OH)_2_ NPs/RGO composite, nanowires were completely transformed into nanoplates (Fig. S2).

**Figure 2:**
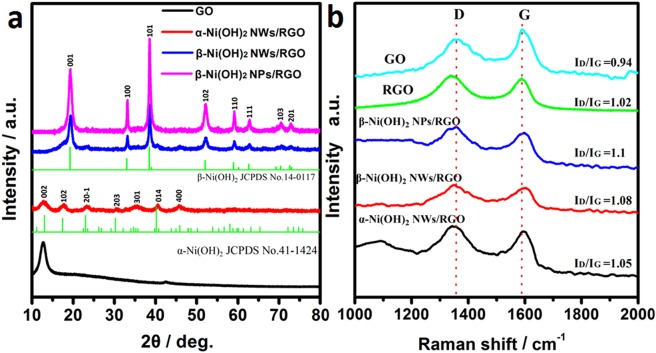
(**a**) XRD patterns of GO, α-Ni(OH)_2_ NWs/RGO, β-Ni(OH)_2_ NWs/RGO, β-Ni(OH)_2_ NPs/RGO. (**b**) Raman spectra of GO, RGO, α-Ni(OH)_2_ NWs/RGO and β-Ni(OH)_2_ NWs/RGO.

Figure 2a shows the XRD patterns of GO and Ni(OH)_2_/RGO composites. For GO, there was a characteristic diffraction peak at 2*θ* = 12.5°, indicating the successful oxidation of the spectral pure graphite^36^. In the Ni(OH)_2_/RGO composites, no characteristic peaks from RGO was observed in XRD patterns, which may be attributed to the ultrathin layer of RGO and strong diffraction peaks of Ni(OH)_2_ ^37^. The main characteristic peaks of α-Ni(OH)_2_ NWs/RGO are corresponded to paraotwayite-type α-Ni(OH)_2_ (JCPDS 41-1424)^38^. After hydrothermal treated, the characteristic peaks of α-Ni(OH)_2_ disappeared, and eventually converted to β-Ni(OH)_2_ (JCPDS 14-0117)^39^. β-Ni(OH)_2_ NPs/RGO had good crystallinity compared with other samples. The weight contents of Ni(OH)_2_ in α-Ni(OH)_2_ NWs/RGO and β-Ni(OH)_2_ NWs/RGO are approximately 95% and 90%, respectively, as calculated from thermogravimetric analysis (TGA) curves (Fig. S3).

As the hydrothermal time increases, the phase and morphology of the Ni(OH)_2_ also changes. In alkaline solution, the anion between the layers of α-Ni(OH)_2_ can be removed after aging, and the α phase of the Ni(OH)_2_ is converted into the β phase. In the absence of alkaline solution, the crystallization rate of β-Ni(OH)_2_ is slower than the dissolution rate of α-Ni(OH)_2_. However, in the NaOH solution, the dissolution rate of α-Ni(OH)_2_ decreases, and the crystallization rate of β-Ni(OH)_2_ increases^35^. Therefore, α-Ni(OH)_2_ can be completely converted to β-Ni(OH)_2_ at 180 °C for 30 min in an alkaline solution. After completely converting β-Ni(OH)_2_ NWs, the morphological changes of β-Ni(OH)_2_ are related to agglomeration and Ostwald ripening^40^. To reduce the surface energy of the crystal, Ni(OH)_2_ nanowires are aggregated into wider nanowires after complete phase transformation. As the hydrothermal time increases, Ni(OH)_2_ nanowires in the defective area break and form nanoplates.

To further confirm the existence and reduction degree of RGO in the composite, we use Raman spectra to characterize the sample. Figure 2b shows that these characteristic D and G peaks of these samples are located at about 1350 and 1590 cm^−1^, respectively. The D peak is associated with first-order zone boundary phonons, indicating the presence of graphene defects. The G peak is attributed to the first-order scattering of the E_2g_ vibrational mode, indicating the in-plane bond-stretching motion of the pairs of C sp^2^ atoms^41^. Generally, the *I*_D_/*I*_G_ ratio is used to determine the reduction level of RGO^42^. The values of RGO (1.02), α-Ni(OH)_2_ NWs/RGO (1.05), β-Ni(OH)_2_ NWs/RGO (1.08), and β-Ni(OH)_2_ NWs/RGO (1.1) are higher than that of GO(0.94), indicating that GO is deeply reduced to form a high-level disordered structure of RGO.

**Figure 3:**
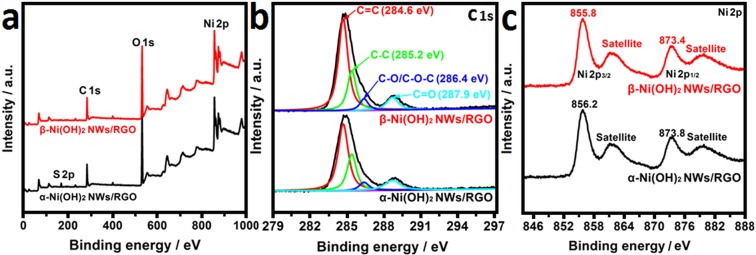
(**a**) XPS survey spectrum of α-Ni(OH)_2_ NWs/RGO and β-Ni(OH)_2_ NWs/RGO, (**b**) C1s and (**c**) Ni 2p XPS spectra of the obtained α-Ni(OH)_2_ NWs/RGO and β-Ni(OH)_2_ NWs/RGO.

XPS analysis is employed to further identify the elemental valence states of Ni(OH)_2_/RGO samples. From survey spectrum of the α-Ni(OH)_2_ NWs/RGO, the C 1s, O 1s, Ni 2p and S 2p peak can clearly be seen (Fig. 3a), but the S 2p peak can not be observed in β-Ni(OH)_2_ NWs/RGO, suggesting α-Ni(OH)_2_ NWs is transformed into β-Ni(OH)_2_ NWs during the hydrothermal reaction in NaOH solution. The C 1s spectrum of XPS is shown in Fig. 3b. The C 1s peak of the Ni(OH)_2_/RGO composites is divided into C=C (284.6 eV), C-C (285.2 eV), C-O/C-O-C (286.4 eV), and C=O (287.9 eV) peaks. The intensity peaks of C=C and C-C bonds in β-Ni(OH)_2_ NWs/RGO are stronger than that of α-Ni(OH)_2_ NWs/RGO, demonstrating that GO was further reduced during the hydrothermal reaction process^43^. The Ni 2p spectrum of XPS is shown in Fig. 3c. The spin separation energy of Ni(OH)_2_ in α-Ni(OH)_2_ NWs/RGO and β-Ni(OH)_2_ NWs/RGO between Ni 2p1/2 (873.4 eV) and Ni 2p3/2 (855.8 eV) is 17.6 eV, which confirm the presence of Ni(OH)_2_ in the composite^44^. The binding energy of α-Ni(OH)_2_ NWs/RGO is higher than that of β-Ni(OH)_2_ NWs/RGO, which is attributed to the presence of SO_4_^2−^ in α-Ni(OH)_2_ NWs.

**Figure 4:**
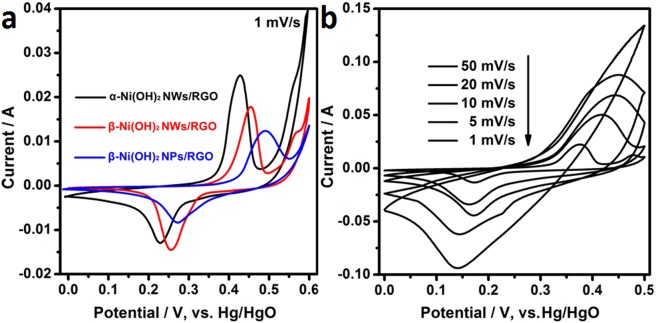
(**a**) CV curves of α-Ni(OH)_2_ NWs/RGO, β-Ni(OH)_2_ NWs/RGO, and β-Ni(OH)_2_ NPs/RGO. Scan rate: 1 mV/s, (**b**) CV curves of β-Ni(OH)_2_ NWs/RGO at different scan rates.

Figure 4a displays the CV profiles of Ni(OH)_2_-based with RGO electrodes at 1 mV/s. A couple of well-defined redox peaks is seen from the CV curves due to the conversion of nickel ions at different oxidation states. The Faradaic reactions of Ni(OH)_2_ electrode is based on the following redox reaction: $N{\rm{i}}{(OH)}_{2}+O{H}^{-}\leftrightarrow N{\rm{i}}OOH+{H}_{2}O+{e}^{-}$ ^45^. The potential difference (*E*_O_ − *E*_R_) between oxidation potential (*E*_O_) and reduction potential (*E*_R_) is used to represent the reversibility of the electrode material. In Table 1, the reversibility of α-Ni(OH)_2_ NWs/RGO and β-Ni(OH)_2_ NWs/RGO is better than that of β-Ni(OH)_2_ NPs/RGO, because the *E*_O_ − *E*_R_ value ranked in the order of α-Ni(OH)_2_ NWs/RGO (0.197 V) < β-Ni(OH)_2_ NWs/RGO (0.2 V) < β-Ni(OH)_2_ NPs/RGO (0.264 V). This is because Ni(OH)_2_ nanowires can provide faster transfer channels for ions and electrons compared to Ni(OH)_2_ nanosheets. For different electrodes, with the same scan speed and active material mass, the larger the CV curve area of the electrode is, the larger the discharge capacity is. It can be concluded that the specific capacities of α-Ni(OH)_2_ NWs/RGO and β-Ni(OH)_2_ NWs/RGO are higher than that of β-Ni(OH)_2_ NPs/RGO at the same test conditions.

**Table 1:** Electrochemical parameters from the CV curves of electrode materials.

Electrode materials	*E*_R_/V	*E*_O_/V	*E*_O_ − *E*_R_/V
α-Ni(OH)_2_ NWs/RGO	0.229	0.426	0.197
β-Ni(OH)_2_ NWs/RGO	0.255	0.455	0.2
β-Ni(OH)_2_ NPs/RGO	0.272	0.491	0.219

The CV plots of β-Ni(OH)_2_ NWs/RGO electrode at different scan rates are shown in Fig. 4b. As the scan rate increases, the oxidation peak moves to the positive direction and the reduction peak moves to the negative direction, and the E_O_ − E_R_ value is constantly increasing, due to the polarization of β-Ni(OH)_2_ NWs/RGO electrode. Furthermore, β-Ni(OH)_2_ NWs/RGO has well-defined redox peaks at a scan rate of 1–20 mV/s, indicating β-Ni(OH)_2_ NWs/RGO has pseudocapacitive properties.

**Figure 5:**
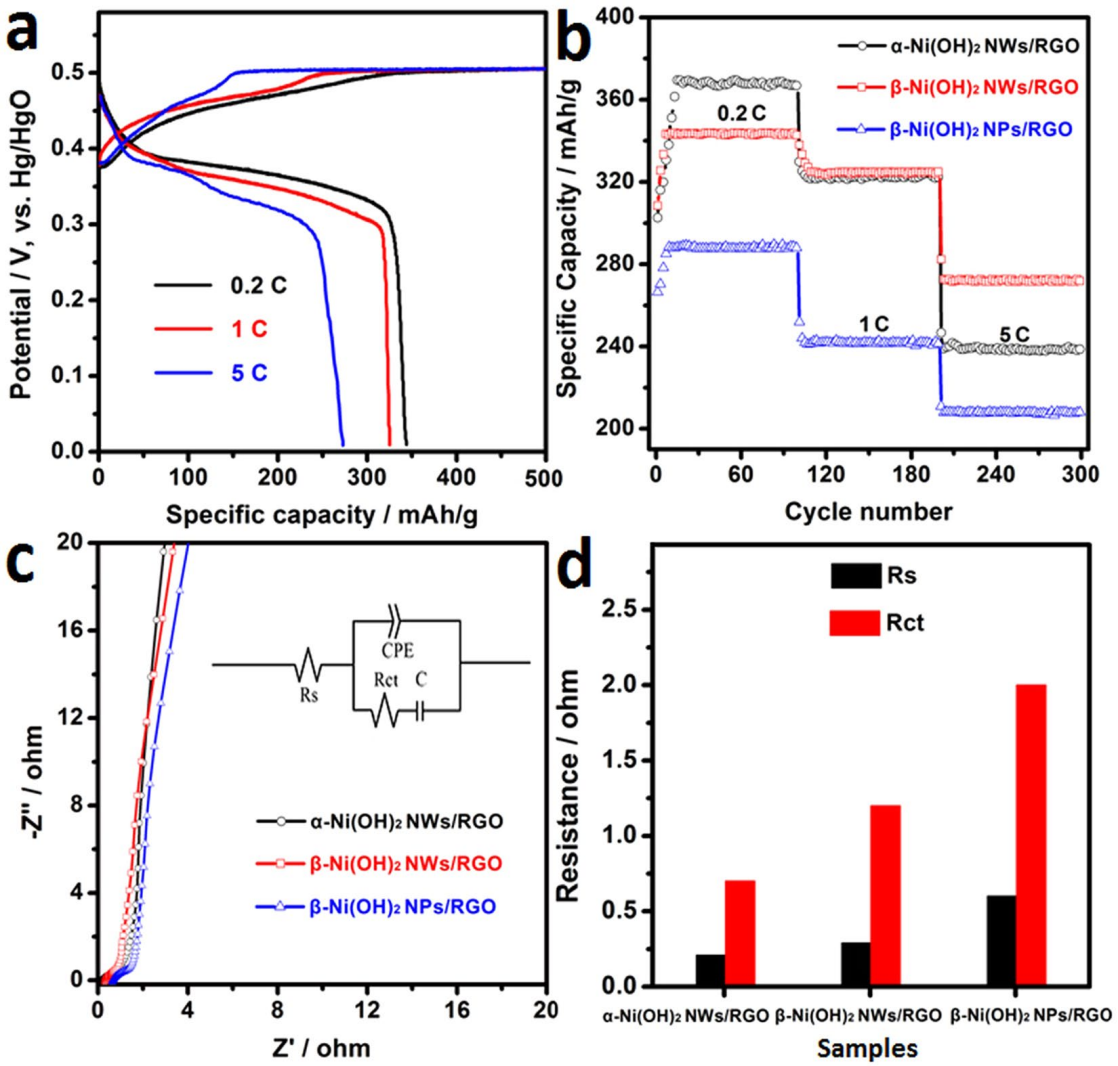
(**a**) Charge-discharge curves of β-Ni(OH)_2_ NWs/RGO at different rates, (**b**) Cycling stability curves of α-Ni(OH)_2_ NWs/RGO, β-Ni(OH)_2_ NWs/RGO, and β-Ni(OH)_2_ NPs/RGO, (**c**) Nyquist plots of α-Ni(OH)_2_ NWs/RGO, β-Ni(OH)_2_ NWs/RGO and β-Ni(OH)_2_ NPs/RGO, (**d**) the fitting value of Rs and Rct resistance.

The charge-discharge performance of β-Ni(OH)_2_ NWs/RGO at various charge-discharge rates is shown in Fig. 5a. As the charge-discharge rate increases, the discharge capacity of the electrode material decreases due to the polarization of the electrode. The discharge specific capacity is 343.2, 325 and 272.7 mAh/g, when the discharge rate is 0.2, 1 and 5C, respectively. Due to synergistic effects of β-Ni(OH)_2_ NWs and RGO, β-Ni(OH)_2_ NWs/RGO shows high capacity and good rate performance, which is larger than reported in some literatures (Table S1). The addition of graphene greatly improved the electrochemical performance of Ni(OH)_2_, which is similar to the graphene-based composites reported in some literature^46–48^.

Figure 5b shows the cycling stability plots of Ni(OH)_2_/RGO electrode at various discharge rates. The average discharge capacities for α-Ni(OH)_2_ NWs/RGO, β-Ni(OH)_2_ NWs/RGO and β-Ni(OH)_2_ NPs/RGO are 368, 343.5 and 288 mAh/g at the discharge rate of 0.2C, and decrease to 238.9, 272.1 and 207.9 mAh/g at the discharge rate of 5C, respectively. This is related to the fact that Ni(OH)_2_ nanowires can provide fast transfer channels for ions and electrons compared to Ni(OH)_2_ nanosheets. After 100 cycles, the β-Ni(OH)_2_ NWs/RGO has almost no capacity loss at the discharge rate of 5C, suggesting that the composite has a good cycling durability. This is ascribed to the synergistic effects of β-Ni(OH)_2_ NWs and RGO.

Figure 5c shows the resistance characteristics of the Ni(OH)_2_/RGO electrode were investigated by EIS measurements. In the medium frequency range, the diffusion of the three electrode materials in the electrolyte is not much difference. In the inset, the intrinsic solution resistance (*R*_S_) and the charge transfer resistances (*R*_ct_) are corresponding to the intercept on the real axis and the diameter of the flat semicircle in the high frequency range, respectively^49^. In Fig. 5d, Ni(OH)_2_/RGO electrodes with 3D network-like have smaller *R*_S_ and *R*_ct_ values compared with β-Ni(OH)_2_/RGO with nanosheets. Apparently, β-Ni(OH)_2_ NWs/RGO composite is an ideal positive material for high performance Ni-MH batteries.

## Conclusion

In summary, we have successfully prepared 3D β-Ni(OH)_2_ NWs/RGO composite via a simple phase transformation method. In NaOH aqueous solution, Paraotwayite type α-Ni(OH)_2_ nanowires have completely transformed into β-Ni(OH)_2_ nanowires when the hydrothermal time was 30 min, and its morphology was still unchanged. The capacity and cycling durability of β-Ni(OH)_2_ NWs/RGO composite was superior to α-Ni(OH)_2_ NWs/RGO composite and β-Ni(OH)_2_ NPs/RGO composite at high discharge rate. Owing to synergistic effects of nanowires and RGO layer, β-Ni(OH)_2_ NWs/RGO composite exhibited high capacity performance and excellent stability.

## Methods

### Preparation of 3D α-Ni(OH)_2_ NWs/RGO composite

GO was prepared by the modified Hummers method. 100 mg GO was dispersed into 60 mL of deionized water via ultrasonic bath for 2 h to form homogeneous GO suspension. NiSO_4_·6H_2_O aqueous solution (20 mL, 0.88 M) and NaOH aqueous solution (20 mL, 0.88 M) was slowly and sequentially dropped into GO suspension under vigorous stirring, and stirred for 10 min. The as-formed suspension was sealed into a 150 mL autoclave. After reaction 120 °C for 24 h, 3D α-Ni(OH)_2_ NWs/RGO composite was collected by vacuum filtration, washed three times with deionized water and absolute ethanol, and then freeze-dried for 24 h.

### Preparation of 3D β-Ni(OH)_2_ NWs/RGO and β-Ni(OH)_2_ NPs/RGO composites

160 mg 3D α-Ni(OH)_2_ NWs/RGO composite was dispersed into NaOH aqueous solution (80 mL, 0.025 M) with vigorous stirring for 30 min. Afterwards, the reaction solution was sealed into a 100 mL autoclave and heated to 180 °C for 30 min and 60 min to synthesis 3D β-Ni(OH)_2_ NWs/RGO and β-Ni(OH)_2_ NPs/RGO composites, respectively. To obtain the final product, the obtained mixture was filtrated and washed with deionized water and absolute ethanol, and then freeze-dried for 24 h.

The crystal structures of prepared samples were characterized by Bruker D8 Advance X-ray diffraction (XRD) with Cu Kα radiation (*λ* = 0.15406 nm); The morphologies and microstructures of prepared samples were observed by field-emission scanning electron microscope (FESEM, Carl Zeiss Sigma 500) and high-resolution transmission electron microscopy (HRTEM, FEI Tecnai G^2^ F20 S-TWIN). X-ray photoelectron spectroscopy (XPS, Thermo Scientific, ESCALAB 250 XI), Infrared spectrum (IR, NICOLET 380), and Raman spectroscopy (Bruker, SENTERRA) measurements were employed to study the physical properties of the samples. The active materials, conducting acetylene black and polytetrafluoroethylene (weight ratio of 8:1:1) were added into ethanol solution. The slurry was fabricated and loaded onto the 1 * 1 cm^2^ nickel foam, the working electrodes were obtained. The load mass of each electrode is approximately 2 mg/cm^2^. The electrochemical data was measured by using CT2001A cell test station (LANHE, China) and CHI760E electrochemical workstation (Chenhua, China) under a three-electrode cell configuration at room temperature in 6 M KOH.

## Supplementary information

Supplementary Info
