# Supplementary material for: 3D β-Ni(OH)2 nanowires/RGO composite prepared by phase transformation method for superior electrochemical performance
Source: Sci Rep. 2019 Jul 25;9:10838. doi: 10.1038/s41598-019-47120-9 (PMC6658499; doi:10.1038/s41598-019-47120-9)
Supplement: Supplementary file 1 — Supplementary Info [file 41598_2019_47120_MOESM1_ESM.pdf]

3D β-$Ni(OH)_2$ nanowires/RGO composite prepared by phase transformation method for superior electrochemical performance

Wenxiu He[1,*], Xingsheng Li[1], Shengli An[1,*], Tongjun Li[2], Yongqiang Zhang[1], Jinlong Cui[1]

[1]*School of Chemistry Chemistry and Chemical Engineering, Inner Mongolia University of Science and Technology, Baotou 014010, China.*

[2]*College of Physics and Material Science, Henan Normal University, Xinxiang 453007, China*

E-mail address: nmghwx@sina.com or shengli_an@126.com.

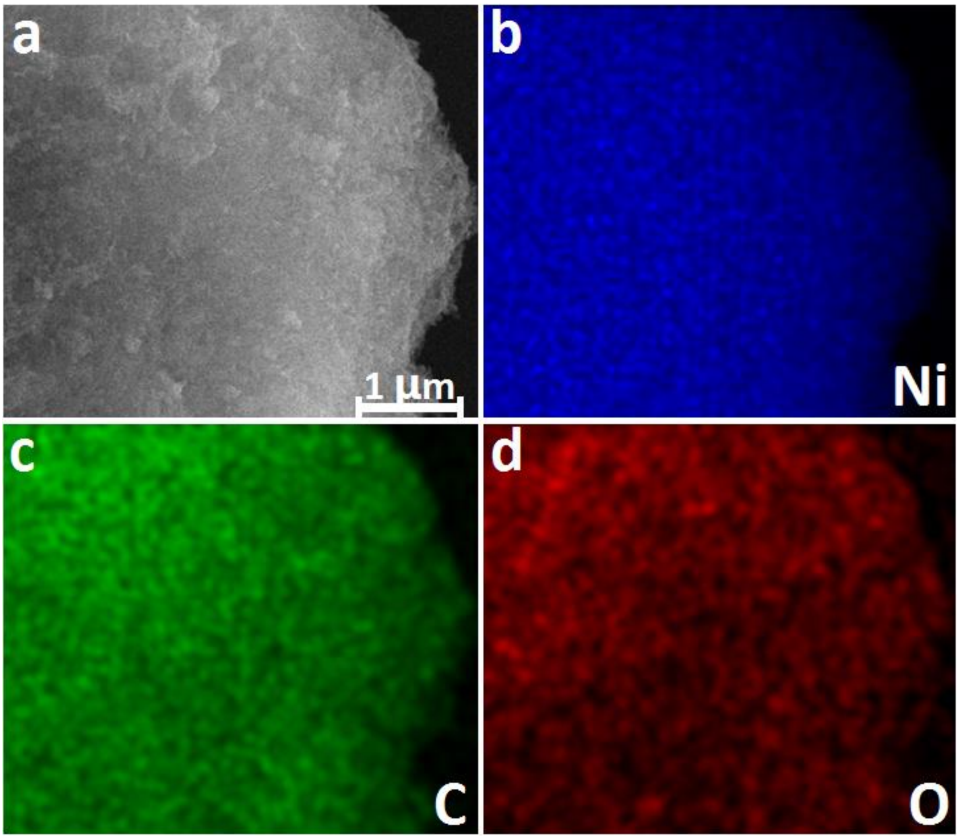

Figure S1. (a) SEM images of β-$Ni(OH)_2$ NWs/RGO and (b) EDS mappings of (c) Ni, (d) O, (e) C of β-$Ni(OH)_2$ NWs/RGO.

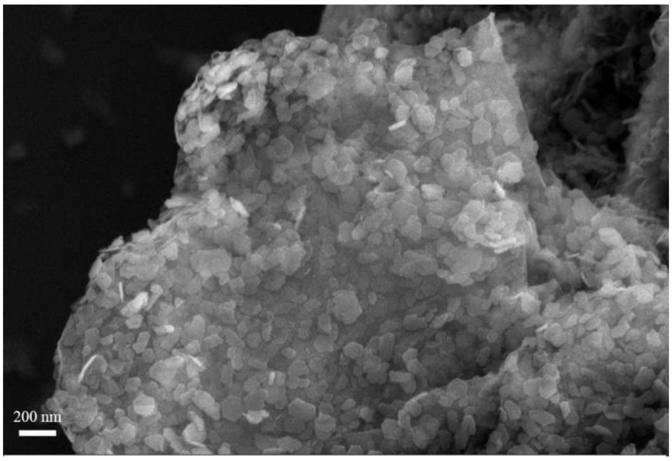

Figure S2. SEM images of β-$Ni(OH)_2$ NPs/RGO.

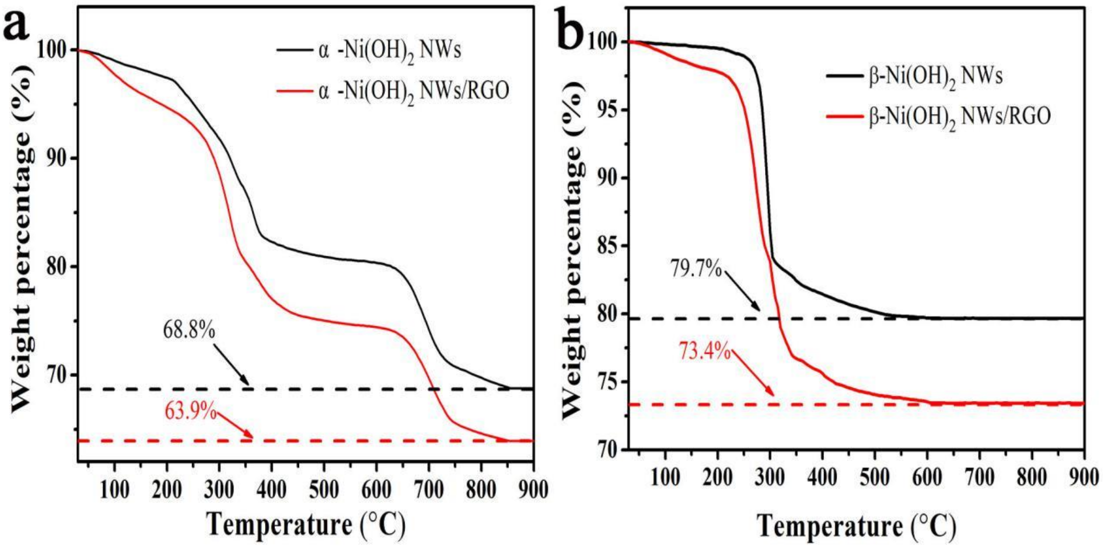

Figure S3. (a) Thermogravimetric analysis (TGA) curves of α-$Ni(OH)_2$ NWs and α-$Ni(OH)_2$ NWs/RGO, (b) TGA) curves of β-$Ni(OH)_2$ NWs and β-$Ni(OH)_2$ NWs/RGO.

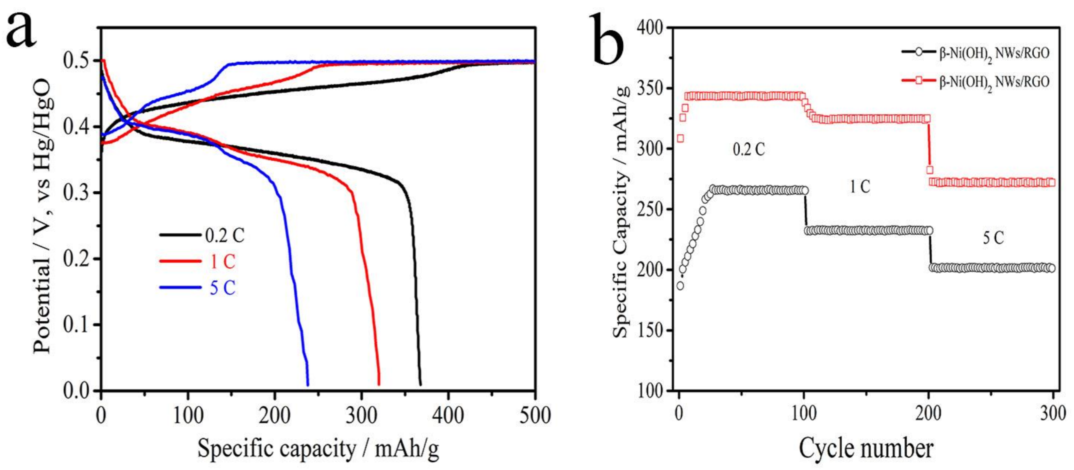

Figure S4. (a) Charge-discharge curves of α-Ni$(OH)_2$ NWs/RGO at different rates, (b) Cycling stability curves of β-Ni$(OH)_2$ NWs and β-Ni$(OH)_2$ NWs/RGO.

The discharge capacity of α-Ni$(OH)_2$ NWs/RGO is significantly higher than that of β-Ni$(OH)_2$ NWs/RGO at 0.2 C, which is related to the fact that the theoretical capacity of α-Ni$(OH)_2$ is higher than that of β-Ni$(OH)_2$ and the structure of α-Ni$(OH)_2$ is not significantly changed at low discharge rate. However, the discharge capacity of β-Ni$(OH)_2$ NWs/RGO is obviously higher than that of α-Ni$(OH)_2$ NWs/RGO at 1C and 5C, which is attributed to the structural stability of β-Ni$(OH)_2$ being higher than the structural stability of α-Ni$(OH)_2$ at high discharge rates (Fig. S4 a). The capacity values of β-Ni$(OH)_2$ NWs/RGO is 343.2 and 288 mAh/g at a discharge rate of 0.2 C, and the values decrease to 272.1 at 5 C. The capacity of β-Ni$(OH)_2$ NWs is 272 mAh/g at 0.2 C, and the values decrease to 206 mAh/g at 5 C. Rate capability of β-Ni$(OH)_2$ NWs/RGO (79.3%) is more than that of β-Ni$(OH)_2$ NWs (75.8%), as shown in Fig. S4 b. The addition of RGO can effectively enhance the conductivity of Ni$(OH)_2$ NWs, relieve its volume expansion during charging-discharging process, and further improve the capacity and cycle stability of Ni$(OH)_2$.

Table S1. Discharge capacities of some β-$Ni(OH)_2$ cathode materials at low discharge rate.

Samples	Discharge rate	Discharge capacities	Ref.
β-$Ni(OH)_2$ NPs	50 mA/g	250.3 mAh/g	[S1]
β-$Ni(OH)_2$ NWs	0.2 C	266.8 mAh/g	[S2]
β-$Ni(OH)_2$/RGO	60 mA/g	283 mAh/g	[S3]
β-$Ni(OH)_2$ NWs/RGO	0.2 C	343.5 mAh/g	This work

Refrences

S1 Li, Y. W., Yao, J. H., Zhu, Y. X., Zou, Z. G., Wang, H. B. Synthesis and electrochemical performance of mixed phase α/β Nickel hydroxide. *J. power sources* **203**, 177-183, https://doi.org/10.1016/j.jpowsour.2011.11.081 (2012).

S2 Li, X. S. et al. Preparation by Phase Transformation Method and Electrochemical Performance of beta-$Ni(OH)_2$ Nanowires. *Chemical Journal of Chinese Universities* **38**, 261-266, https://doi.org/10.7503/cjcu20160511 (2017).

S3 Li, B. J. *et al.* Improved performances of β-$Ni(OH)_2$@reduced-graphene-oxide in Ni-MH and Li-ion batteries. *Chem. Commun.* **47**, 3159–3161, https://doi.org/10.1039/c0cc04507a (2011).
